# Removal of broken abutment screws using ultrasonic tip – a heat development in-vitro study

**DOI:** 10.1186/s12903-023-03654-z

**Published:** 2024-01-02

**Authors:** Vasilios Alevizakos, Anna-Lena Bergmann, Constantin von See

**Affiliations:** Research Center for Digital Technologies in Dentistry and CAD/CAM, Danube Private University, Steiner Landstraße 124, Krems, an der Donau 3500 Austria

**Keywords:** Dental implants, Abutment screws, Heat generation, Screw removal, Tissue damage

## Abstract

**Background:**

Dental implants can cause complications, including the loosening of the abutment screw or fracture. However, there is no standardized technique for removing broken abutment screws. This necessitates further research.

**Objective:**

This study aimed to measure heat generation during screw removal to better understand its implications for dental implant procedures.

**Material and methods:**

The experimental setup involved using synthetic bone blocks and titanium implants. An ultrasonically operated instrument tip was utilized for screw removal. Infrared thermometry was employed for accurate temperature measurement, considering factors such as emissivity and distance. Statistical analysis using linear regression and ANOVA was conducted.

**Results:**

The findings revealed an initial rapid temperature increase during the removal process, followed by a gradual decrease. The regression model demonstrated a strong correlation between time and temperature, indicating the heat generation pattern.

**Conclusion:**

Heat generation during screw removal poses risks such as tissue damage and integration issues. Clinicians should minimize heat risks through an intermittent approach. The lack of a standardized technique requires further research and caution. Understanding the generated heat optimizes implant procedures.

## Introduction

Scientific evidence strongly supports the use of dental implants as an effective solution to restore the function of a missing tooth [1–3]. However, it’s essential to acknowledge that dental implants are not without risks and can lead to inherent biological and mechanical complications, especially concerning inflammatory diseases of peri-implant tissue and screw loosening [4–6]. Among these complications, the rupture and loosening of the abutment screw in implant-supported restorations have been identified as the most common issues [7–9].

The occurrence of abutment screw fractures has been reported to vary between 0 and 10.4% in studies with a 5-year follow-up, with Jung et al. reporting an incidence of screw loosening of 12.7% after a 5-year follow-up in implant-supported single crowns [10–13]. More recently, a study demonstrated that abutment screw loosening ranged between 7 and 11%, while others found that 25% of patients experienced screw loosening during routine follow-up [14, 15]. The problem of screw loosening and breakage is widespread and significantly affects the restorative aspect of dental implants. The main causes are often inadequate biomechanical design and/or occlusal overload, leading to eventual screw fracture [16]. In addition to screw loosening, in-vitro studies had pointed out that inadequate biomechanical design and occlusal overload play crucial roles in influencing the fatigue and fracture performance of implants. The study by Cosola et al. highlighted that the presence of a ferrulized neck in a single implant-supported screw-retained crown results in significant differences compared to other systems. Various factors, such as abutment collar height, abutment diameter, and the type of crown retention, exert significant influence on the strength and behavior of implant systems under cyclic loading conditions [17].

Addressing the issue of broken abutment screws poses a significant challenge as it requires careful and time-consuming treatment, with varying degrees of risk for both the implant and prosthesis that need to be thoroughly assessed [18]. The primary goal of treatment when an abutment screw breaks is to remove the fragment without causing damage to the internal thread of the implant and implant shoulder, subsequently replacing the broken screw with a new one. Unfortunately, there is no standardized technique for removing broken abutment screws, leading to the existence of numerous techniques and devices [19, 20].

Among the methods employed for removing broken abutment screws, Imam et al. used stainless steel probes and instruments attached to a handpiece at low speed, with fractures in the apical part of the implant’s internal thread requiring the option of ultrasound instruments if hand instruments prove unsuccessful [21]. Despite the availability of multiple methods, the conventional approach involving the use of a probe and ultrasound remains a highly efficient and economical choice for removing fractured abutment screws, supported by statistical data indicating a removal success rate of 73.3% [22].

In cases where removing the broken abutment screw becomes unfeasible, the dental implant must be removed, leading to increased costs and time requirements when a new dental implant needs to be placed. However, Kim et al. suggested an alternative approach, proposing the replacement of clinically broken abutment screws with shorter ones without removing the remnants of the broken screw [23, 24].

It’s important to consider the potential risks associated with the ultrasonic approach used during the removal procedures of broken abutment screws. Ultrasound refers to sound waves with frequencies higher than the upper limit of human hearing, typically above 20,000 hertz (Hz). When ultrasound waves propagate through a medium like metal, they can interact in various ways, including generating heat through mechanical vibrations at the atomic or molecular level. This phenomenon is known as ultrasonic heating. The heat generated during the removal process of broken abutment screws can have irreversible effects on the survival of peri-implant tissue and may affect the osseointegration of the dental implant. Kniha et al. indicated that a temperature threshold between 47 and 55 °C could cause bone necrosis [25].

Thus, the aim of the present study is to measure the heat generated on the implant surface during the removal of screw fragments. By carefully investigating this aspect, we can gain valuable insights into the potential risks and take necessary precautions to ensure long-term success of dental implant treatments.

## Materials and methods

The study aimed to investigate the heat generation on the collar region of titanium implants (Ø4.0 mm L9.5 mm, SIC-Invent, Basel, Switzerland) during the removal of broken abutment screws using an ultrasonically operated instrument tip (CAVI 2-D, VDW. ULTRA, VDW GmbH, Munich, Germany).

**Fig. 1:**
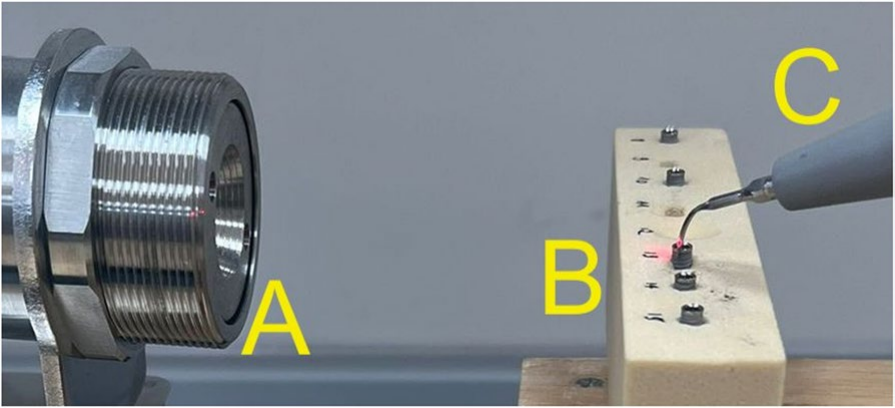
Ultrasonic-driven tip used for screw fragment removal. An infrared thermometer; B specimen to be measured; C ultrasonic-driven instrument

Ten implants were inserted supracrestal into a synthetic bone block (#1522–12 — 20 PCF, Sawbone, A Pacific Research Company, Vashon Island, Washington, USA) up to 2 mm supracrestal (Fig. 1). To ensure a comparable situation of screw fracture, the abutment screws were weakened by grinding the diameter of the screws at a predetermined breaking point at the beginning of the thread (2 mm), which broke upon screw tightening. The screw removal was carried out using the “MAXI” mode on the device (VDW ULTRA, VDW GmbH, Munich, Germany), specifically designed for fragment removal, and conducted by two experienced clinicians.

**Fig. 2:**
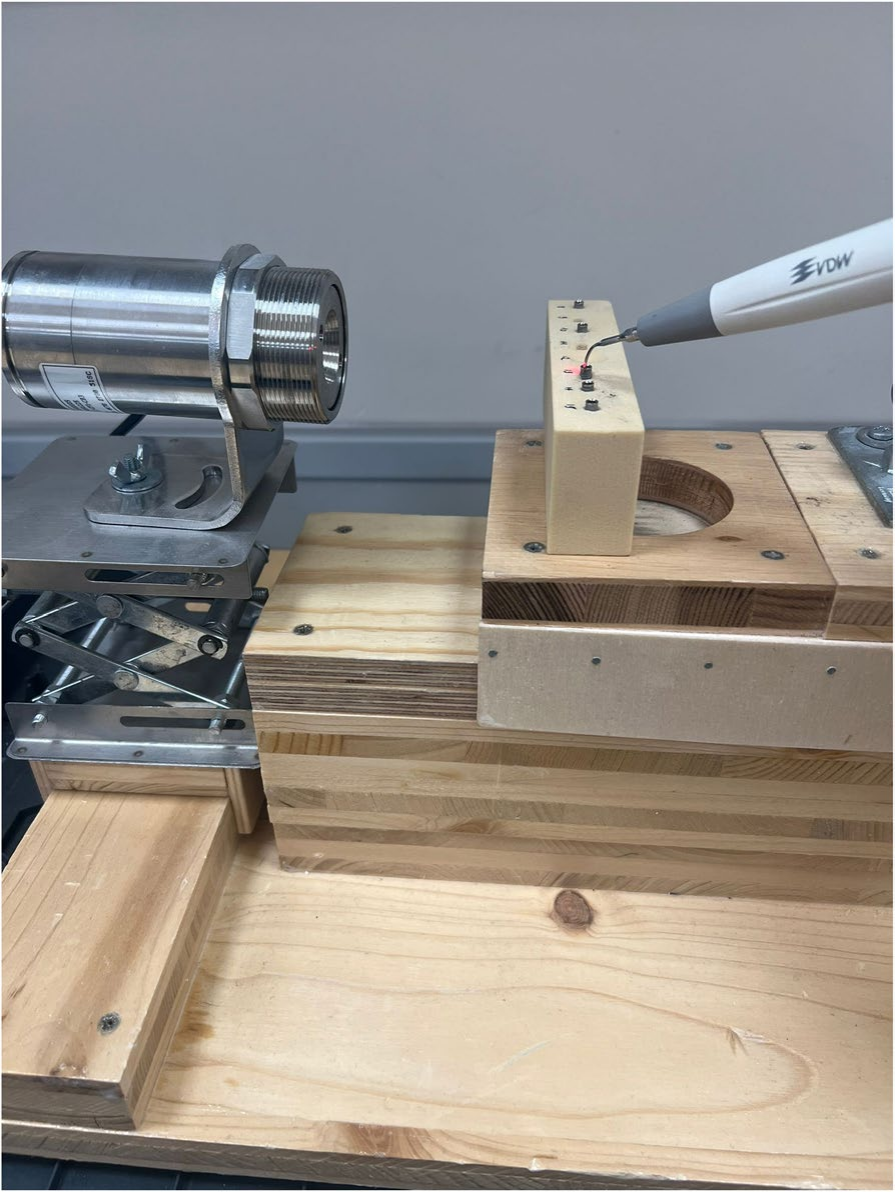
Experimental set-up. The laser points at the area to be measured

For temperature measurements, an infrared thermometer (thermoMETER CTL, Micro-EPSILON®, MICRO-EPSILON MESSTECHNIK GmbH & Co. KG, Ortenburg, Germany) was utilized. Since infrared thermometers have limitations when measuring the temperature of metallic surfaces due to unreliable emissivity, the implant’s surface was coated with paint, and its specific emissivity was calculated. This involved preliminary tests where the painted implant was brought to room temperature through a two-hour acclimatization period, and the room temperature was determined using accurate thermometers. The specific emissivity for the implant was then set at 0.95 using the infrared thermometer software (CompactConnect software from MICRO-EPSILON®). The infrared thermometer measures the infrared radiation emitted by the measuring object and calculates the surface temperature on this basis. Via an integrated double laser sighting, the measuring spot is precisely marked in size and position on the object’s surface. The measuring spot size is 0.9 mm at 7 cm. The infrared thermometer was placed on a movable platform with adjustable height and a movable carriage for fine adjustments, allowing precise targeting of the 2 mm supracrestal implant shoulder. The measurement commenced at the beginning of fragment removal (Fig. 2), and the temperature changes were recorded and subjected to statistical analysis. The infrared thermometer software recorded the temperature changes by time in seconds. A linear regression analysis was performed to assess the influence of the variable “time in seconds” on the variable “temperature in degrees Celsius.”

## Results

**Fig. 3:**
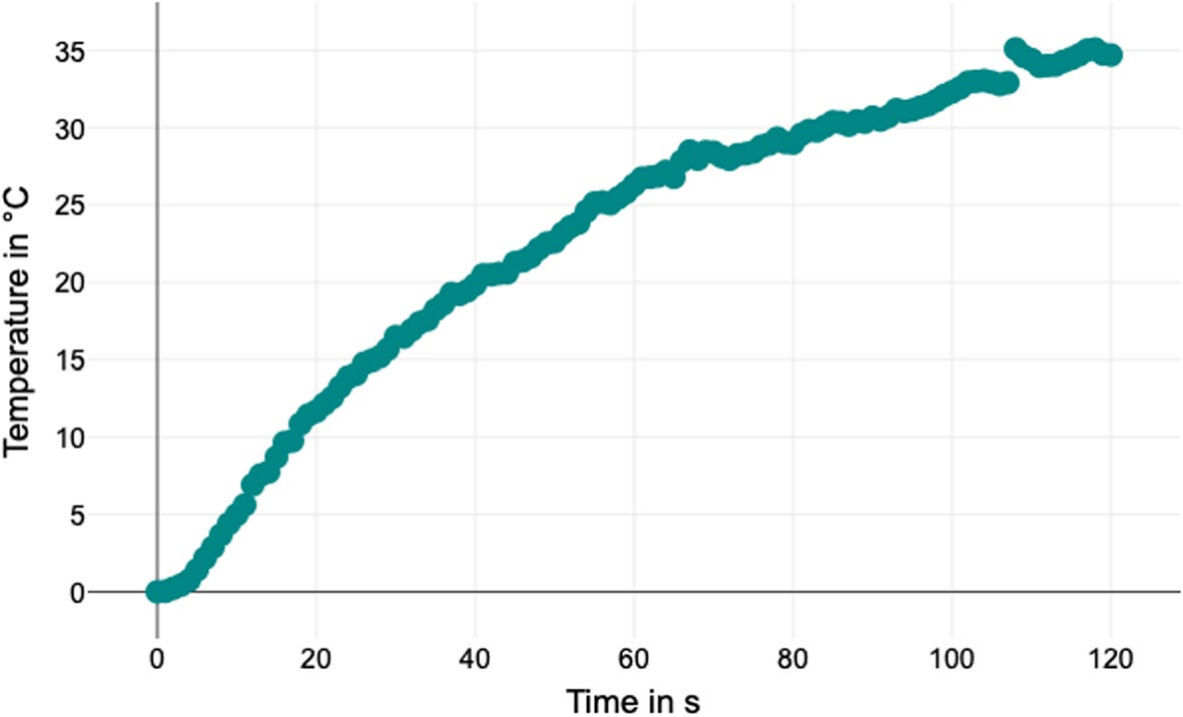
Regression analysis displayed on a graph

The temperature change exhibited a notable increase during the initial 10 seconds, gradually tapering off thereafter. At 60 seconds, the temperature reached 50.58 °C ± 9.23, and at the end of measurement at 120 seconds, the temperature reached 59.53 °C ± 11.21. A linear regression analysis was conducted to explore the relationship between the variable “time in seconds” and the variable “temperature in degrees Celsius” (Fig. 3). The regression model showed a strong association, with an R2 value of 0.92, indicating that approximately 92.05% of the variance in temperature could be explained by the elapsed time.

The model’s predictive capability for the variable “temperature in degrees Celsius” was assessed with a standard error of estimate of 2.85. Further, an ANOVA was conducted to determine if this value significantly deviated from zero. The analysis showed a highly significant effect (F = 1378.68, *p* < 0.001), confirming the model’s reliability in predicting temperature changes over time, with an R2 of 0.92.

The obtained regression model is as follows:

$$\textbf{Temperature}\ \textbf{in}\ \textbf{degrees}\ \textbf{Celsius}=\textbf{6.24}+\textbf{0.28}\ast \textbf{Time}\ \textbf{in}\ \textbf{seconds}.$$

When all independent variables are zero, the value of the variable “temperature in degrees Celsius” is 6.24. This constant value can be interpreted as the baseline temperature. Additionally, if the value of the variable “time in seconds” changes by one unit, the value of the variable “temperature in degrees Celsius” changes by 0.28.

## Discussion

In this study, it was observed that the removal of screw fragments from the implant resulted in significant heat generation on the implant surface.

To prevent screw loosening and fracture, clinicians should have a good understanding of the mechanics of the abutment screw. When a screw is tightened, it creates a torque, resulting in a force known as preload. Due to the elastic reset of the screw, the two parts are pulled together, generating a clamping force. The preload in the screw, arising from elongation and elastic return, equals the clamping force [26]. It has been reported that 2 to 10% of the initial preload is lost as a result of setting.

To minimize the settling effect, abutment screws should be retightened 10 minutes after the initial torque tightening [27, 28]. Additionally, the use of implant components with anti-rotation features and low tolerances for component misfit can help reduce abutment screw loosening and related complications [29].

Unnoticed screw loosening, which is the primary cause of screw fracture, can result from a non-passive implant superstructure, manufacturing defects, and biomechanical overload [30–32]. Nanoscale axial movements resulting from prosthetic loading have also been reported to contribute to abutment screw fractures [27, 33, 34]. While internal connections, anti-rotational, and conical designs can enhance resistance to abutment screw loosening, they may also increase the risk of abutment screw fractures. Huang et al. concluded that screw loosening poses a common challenge in implant-supported restorations, underscoring the importance of understanding its underlying causes and relevant factors for clinicians to make informed decisions in practice. Their comprehensive review offers several key insights. First, internal connections demonstrate superior resistance to torque loss and screw loosening compared to external connections. Second, the geometrical design of the abutment, including features like anti-rotational and conical designs, plays a crucial role in stabilizing the implant-abutment connection. Additionally, original components generally exhibit greater stability than non-original ones. The impact of surface treatment on the abutment screw’s stability in the implant-abutment connection remains a subject of debate. Cantilevers, it is noted, can lead to stress concentration and heighten the risk of screw loosening. Proper tightening procedures are paramount—abutment screws must be secured to the manufacturer’s recommended torque during insertion, with caution against repeated loosening and tightening. Retorquing the screw after initial torquing, with a specified time interval, may help reduce preload loss.

Individual patient variations warrant careful consideration, especially in terms of parafunctional habits like bruxism, excessive occlusal force, and unfavorable chewing patterns. By taking these factors into account, clinicians can proactively address the issue of screw loosening in implant-supported restorations, ultimately enhancing the longevity and stability of the treatment [35]. The etiology of screw fractures is multifactorial and can include factors such as inadequate treatment planning, misfit of components, inadequate screw tightening, excessive loading, and inadequate screw design [36]. Inadequate treatment design and planning can stem from patient assessment and insufficient number and positioning of implants. Among various restorations, single crowns are more susceptible to screw loosening, followed by cantilevered bridges, blocked crowns, and implant-supported overdentures [37]. Fractures typically occur at the junction between the screw head and shaft or at the junction between the screw shaft and thread [38, 39]. When an abutment screw above the implant head breaks, conservative methods involving the use of an investigator, straight probe, or hemostasis can often be successful [19, 40].

The first step in removing a broken screw is to conduct a detailed medical history and thorough clinical examination to determine the cause of the screw fracture, thereby minimizing the risk of complications. The tip of the instrument should be carefully maneuvered counterclockwise over the surface of the screw segment until it becomes loose. Even with a fracture of the abutment screw below the implant platform, the initial intervention choice involves using rigid instruments such as a scaler, sickle probe, or endodontic probe [41–43]. Ultrasonic oscillation using hand instruments is an additional method to remove a fractured abutment screw fragment, which may not be possible with hand instruments alone [44, 45]. In the present study, an ultrasonic device was used to remove the screw fragments. Studies have shown that ultrasonic devices show promising results in the removal of metallic posts in endodontics [46, 47]. Thin scaler tips that oscillate counterclockwise can be useful in retracting the fragment, but caution should be exercised to avoid wedging the screw further into the implant. Gooty et al. have proposed the successful use of an ultrasonic scaler to remove a broken screw [48]. The long, non-cutting tip of the ultrasonic scaler provides good predictability for this method [44]. Chen and Cho have also suggested the use of a TU17/23 double-ended probe from Hu-Friedy (STERIS plc, Derby, United Kingdom) to rotate the fragment counterclockwise [45]. The Hu-Friedy TU17/23 is a double-ended explorer, the #TU17 end for subgingival calculus detection and the #23 end for caries detection. If these techniques fail, a stiffer handheld scalpelizer can be employed to detect the fracture surface. When the fragment remains unretrievable, an adhesive holder for dentures (True Grip; Clinician’s Choice) can be used to capture the top of the fragment and rotate it first clockwise and then counterclockwise. In cases where conservative methods of removing broken screws are unsuccessful, some authors recommend using commercial extraction kits. Nayana et al. have proposed a novel technique for recovering implant abutment screws broken by excessive torque, using an ultrasonic scaler [49]. The tip of the ultrasonic scaler is curved counterclockwise around the fractured screw, while an assistant guides a sharp, straight probe diagonally opposite the scaler tip over the broken fragment, securing it between the implant body and the fractured screw. In most cases, patients prefer avoiding replacement with a new implant due to the associated expense and requirement for surgical intervention [49]. Although clinicians may use various techniques to remove the fractured abutment screws, every effort should be made to eliminate the cause of the screw fracture.

It should be noted that ultrasound can lead to an increase in temperature, potentially affecting the surrounding periodontal tissue [50]. Studies have also reported morphological damage to bone tissue above 47 °C and permanent damage to bone tissue between 56 and 60 °C, with alkaline phosphatase inactivation occurring at 56 °C, which is considered a critical denaturation temperature [44, 51]. Furthermore, Dominici et al. have reported that endodontic pen removal with ultrasonic vibrations without rinsing also led to temperature increases exceeding 47 °C after 15 seconds [51–53]. Additionally, the use of non-irrigation ultrasound techniques for endodontic pen removal generated sufficient heat at the root surface for 4 minutes to potentially affect neighboring teeth [54].

The study has several important limitations that should be acknowledged. First, it is essential to recognize that this research was conducted in an in-vitro setting, which may not entirely replicate real-world clinical conditions. While in-vitro studies offer controlled environments for focused analysis, they may not capture the full spectrum of variables encountered in actual patient scenarios. Additionally, the number of implants tested in this study was relatively small, which may impact the generalizability of the findings. Furthermore, it’s worth noting that this study specifically examined one brand of materials, and it is possible that different brands or materials could yield different results and implications. Thus, caution should be exercised in extrapolating these findings to broader clinical applications. Future research endeavors should aim to address these limitations and further explore the nuances of implant-supported restorations across a wider range of clinical contexts and materials.

**Fig. 4:**
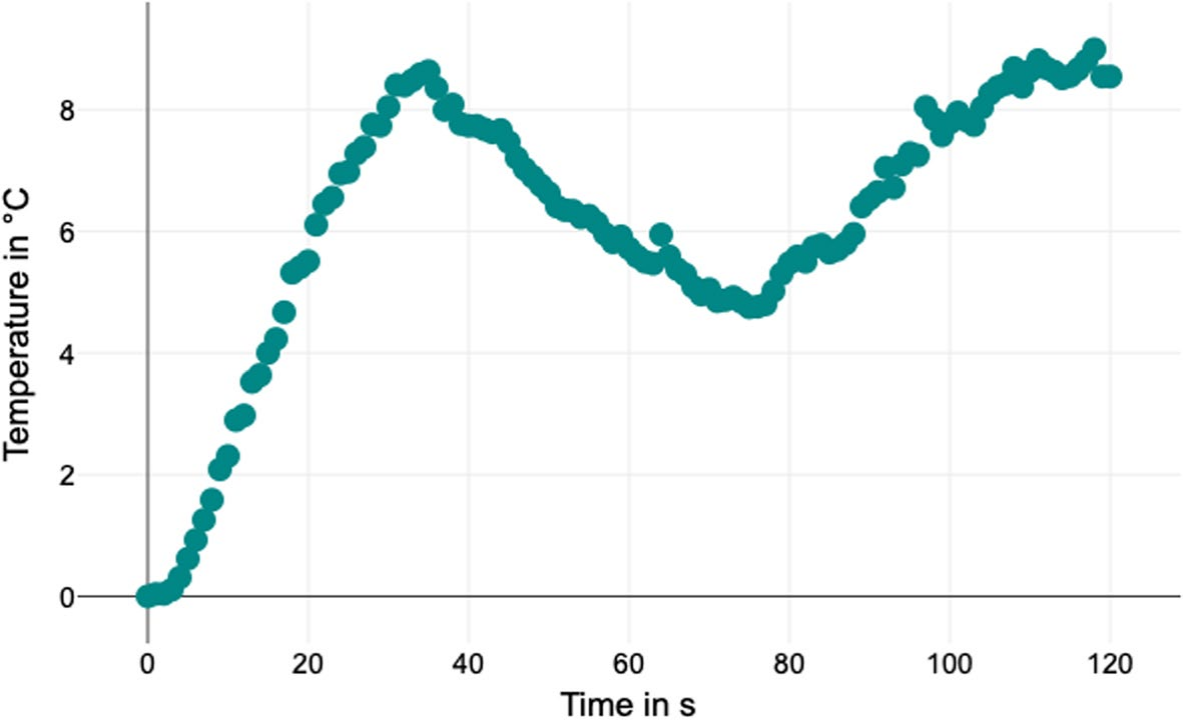
Temperature rise during screw fragment removal – an intermittent approach

Despite these findings, further clinical research is necessary to determine the clinical significance of implant abutment damage and heat generation, as well as the prognosis of dental implants. An intermittent approach may be considered to minimize heat generation. Pausing the removal of the screw fragment when a temperature increase of 10 °C is reached and resuming after cooling could potentially allow for screw removal without harmful overheating (Fig. 4).

## Conclusion

The recovery of the broken screw fragment should be done carefully to avoid internal damage to the implant structure. However, ultrasound technology proved to be more effective for extracting fractured abutment screws. However, overheating of the implant is possible. Thus, an intermittent approach may be advisable.

## Data Availability

The data that support the findings of this study are available from the corresponding author, [VA], upon reasonable request.

## References

[1] Branemark PI (1977). Osseointegrated implants in the treatment of the edentulous jaw: experience from a 10-year period. Scad J Plast Reconstr Surg..

[2] Naert I (1992). A six-year prosthodontic study of 509 consecutively inserted implants for the treatment of partial edentulism. J Prosthet Dent..

[3] Quirynen M, Naert I, Van Steenberghe D (1992). Fixture design and overload influence marginal bone loss and future success in the Brånemark® system. Clin Oral Implants Res..

[4] Renvert S (2018). Peri-implant health, peri-implant mucositis, and peri-implantitis: case definitions and diagnostic considerations. J Periodontol..

[5] Schwarz F (2018). Peri-implantitis. J Clin Periodontol..

[6] Smeets R (2014). Definition, etiology, prevention and treatment of peri-implantitis--a review. Head Face Med..

[7] Berglundh T (2018). Peri-implant diseases and conditions: consensus report of workgroup 4 of the 2017 world workshop on the classification of periodontal and Peri-implant diseases and conditions. J Clin Periodontol..

[8] Lee JH, Cha HS (2018). Screw loosening and changes in removal torque relative to abutment screw length in a dental implant with external abutment connection after oblique cyclic loading. J Adv Prosthodont..

[9] Yoon JH, Lee H, Kim MY (2016). Safe removal of a broken abutment screw with customized Drill guide and rotary instrument: a clinical report. J Prosthodont..

[10] Jung RE (2008). A systematic review of the 5-year survival and complication rates of implant-supported single crowns. Clin Oral Implants Res..

[11] Papaspyridakos P (2012). A systematic review of biologic and technical complications with fixed implant rehabilitations for edentulous patients. Int J Oral Maxillofac Implants..

[12] Sailer I (2012). Cemented and screw-retained implant reconstructions: a systematic review of the survival and complication rates. Clin Oral Implants Res..

[13] Vanlıoğlu B, Özkan Y, Kulak-Özkan Y (2013). Retrospective analysis of prosthetic complications of implant-supported fixed partial dentures after an observation period of 5 to 10 years. Int J Oral Maxillofac Implants..

[14] Katsavochristou A, Koumoulis D (2019). Incidence of abutment screw failure of single or splinted implant prostheses: a review and update on current clinical status. J Oral Rehabil..

[15] Wie H (1995). Registration of localization, occlusion and occluding materials for failing screw joints in the Brånemark implant system. Clin Oral Implants Res..

[16] Winkler S (2003). Implant screw mechanics and the settling effect: overview. J Oral Implantol..

[17] Cosola S (2021). In-vitro fatigue and fracture performance of three different ferrulized implant connections used in fixed prosthesis. J Dent Sci..

[18] Gupta S, Gupta H, Tandan A (2015). Technical complications of implant-causes and management: a comprehensive review. Natl J Maxillofac Surg..

[19] Barbosa JM (2014). The cotton driver: an alternative technique for removing fractured screw fragments. J Prosthet Dent..

[20] Williamson RT, Robinson FG (2001). Retrieval technique for fractured implant screws. J Prosthet Dent..

[21] Imam AY (2014). A technique for retrieving fractured implant screws. J Prosthet Dent..

[22] Agustín-Panadero R (2020). In vitro comparison of the efficacy of two fractured implant-prosthesis screw extraction methods: Conventional versus mechanical. J Prosthet Dent..

[23] Carneiro Tde A (2016). A conservative approach to retrieve a fractured abutment screw – case report. J Prosthodont Res..

[24] Kim BJ (2012). The effect of screw length on fracture load and abutment strain in dental implants with external abutment connections. Int J Oral Maxillofac Implants..

[25] Kniha K (2020). Temperature threshold values of bone necrosis for Thermo-Explantation of dental implants –A systematic review on preclinical in vivo research. Materials..

[26] Abdelfattah YM (2014). Different mechanical complications of implant prosthodontics: review article. Int J Dent Res..

[27] Bakaeen LG, Winkler S, Neff PA (2001). The effect of implant diameter, restoration design, and occlusal table variations on screw loosening of posterior single-tooth implant restorations. J Oral Implantol..

[28] Siamos G, Winkler S, Boberick KG (2002). Relationship between implant preload and screw loosening on implant-supported prostheses. J Oral Implantol..

[29] Cho SC (2004). Screw loosening for standard and wide diameter implants in partially edentulous cases: 3- to 7-year longitudinal data. Implant Dent..

[30] Goodacre CJ, Kan JYK, Rungcharassaeng K (1999). Clinical complications of osseointegrated implants. J Prosthet Dent..

[31] McGlumphy EA, Mendel DA, Holloway JA (1998). Implant screw mechanics. Dent Clin N Am..

[32] Nergiz I, Schmage P, Shahin R (2004). Removal of a fractured implant abutment screw: a clinical report. J Prosthet Dent..

[33] Eckert SE (2000). Analysis of incidence and associated factors with fractured implants: a retrospective study. Int J Oral Maxillofac Implants..

[34] Tagger Green N (2002). Fracture of dental implants: literature review and report of a case. Implant Dent..

[35] Huang Y, Wang J (2019). Mechanism of and factors associated with the loosening of the implant abutment screw: a review. J Esthet Restor Dent..

[36] Raju S (2021). Management of perishing implants with abutment screw fracture – a systematic review. J Indian Prosthodont Soc..

[37] Lee KY (2020). Clinical study on screw loosening in dental implant prostheses: a 6-year retrospective study. J Korean Assoc Oral Maxillofac Surg..

[38] Quek CE, Tan KB, Nicholls JI (2006). Load fatigue performance of a single-tooth implant abutment system: effect of diameter. Int J Oral Maxillofac Implants..

[39] Taira Y, Sawase T (2012). A modified technique for removing a failed abutment screw from an implant with a custom guide tube. J Oral Implantol..

[40] Yang CH, Wu AY (2019). A technique to retrieve a fractured implant abutment screw by using a screwdriver fashioned from a needle. J Prosthet Dent..

[41] Azpiazu-Flores FX, Lee DJ (2020). Using the screw shank as a retrieval tool: a straightforward approach to removing screws with diagonal fractures. J Prosthet Dent..

[42] Fauvell SA, Gialanella G, Penna KJ (2006). The lumen technique. retrieval of broken gold screws in dental implants. N Y State Dent J..

[43] Satterthwaite J, Rickman L (2008). Retrieval of a fractured abutment screw thread from an implant: a case report. Br Dent J..

[44] Bhandari S, Aggarwal N, Bakshi S (2013). Ultrasonic oscillations for conservative retrieval of a rare fracture of implant healing abutment. J Oral Implantol..

[45] Chen JH, Cho SH (2018). An accessory technique for the intraoral removal of a fractured implant abutment screw. J Prosthet Dent..

[46] Johnson WT, Leary JM, Boyer DB (1996). Effect of ultrasonic vibration on post removal in extracted human premolar teeth. J Endod..

[47] Dixon EB (2002). Comparison of two ultrasonic instruments for post removal. J Endod..

[48] Gooty JR (2014). Noninvasive method for retrieval of broken dental implant abutment screw. Contemp Clin Dent..

[49] Nayana P (2022). Retrieval of fractured implant abutment screws: a narrative review. J Int Soc Prev Community Dent..

[50] Davis S (2010). Analysis of temperature rise and the use of coolants in the dissipation of ultrasonic heat buildup during post removal. J Endod..

[51] Huttula AS (2006). The effect of ultrasonic post instrumentation on root surface temperature. J Endod..

[52] Horan BB (2008). Effect of dentin thickness on root surface temperature of teeth undergoing ultrasonic removal of posts. J Endod..

[53] Walters JD, Rawal SY (2007). Severe periodontal damage by an ultrasonic endodontic device: a case report. Dent Traumatol..

[54] Capriotti L (2018). Removal of fiber posts during endodontic retreatments using ultrasonic tips: a comparison between two different endodontic fiber posts. G Ital..

